# Long-term results of edge-to-edge and neochordal mitral repair for isolated anterior leaflet lesion: a propensity match analysis

**DOI:** 10.1093/ejcts/ezae435

**Published:** 2024-12-03

**Authors:** Edoardo Zancanaro, Davide Carino, Roberto Lorusso, Benedetto Del Forno, Elisabetta Lapenna, Alessandra Sala, Guido Ascione, Maria Giovanna Scarale, Alessandro Nonis, Alessandro Castiglioni, Ottavio Alfieri, Francesco Maisano, Michele De Bonis

**Affiliations:** Department of Cardiac Surgery, IRCCS San Raffaele Scientific Institute, Vita-Salute San Raffaele University, Milan, Italy; Department of Cardiac Surgery, IRCCS San Raffaele Scientific Institute, Vita-Salute San Raffaele University, Milan, Italy; Cardio-Thoracic Surgery Department, Heart and Vascular Centre, Maastricht University Medical Centre, Maastricht, Netherlands; Cardiovascular Research Institute Maastricht, Maastricht, Netherlands; Department of Cardiac Surgery, IRCCS San Raffaele Scientific Institute, Vita-Salute San Raffaele University, Milan, Italy; Department of Cardiac Surgery, IRCCS San Raffaele Scientific Institute, Vita-Salute San Raffaele University, Milan, Italy; Department of Cardiac Surgery, IRCCS San Raffaele Scientific Institute, Vita-Salute San Raffaele University, Milan, Italy; Department of Cardiac Surgery, IRCCS San Raffaele Scientific Institute, Vita-Salute San Raffaele University, Milan, Italy; University Centre of Statistics in Biomedical Sciences (CUSSB), Vita Salute San Raffaele University, Milan, Italy; University Centre of Statistics in Biomedical Sciences (CUSSB), Vita Salute San Raffaele University, Milan, Italy; Department of Cardiac Surgery, IRCCS San Raffaele Scientific Institute, Vita-Salute San Raffaele University, Milan, Italy; Department of Cardiac Surgery, IRCCS San Raffaele Scientific Institute, Vita-Salute San Raffaele University, Milan, Italy; Department of Cardiac Surgery, IRCCS San Raffaele Scientific Institute, Vita-Salute San Raffaele University, Milan, Italy; Department of Cardiac Surgery, IRCCS San Raffaele Scientific Institute, Vita-Salute San Raffaele University, Milan, Italy

**Keywords:** Mitral valve repair, Edge to Edge Repair, Mitral valve, anterior mitral leaflets, neochordal repair

## Abstract

**OBJECTIVES:**

Mitral regurgitation due to anterior mitral leaflet lesions is associated with an increased risk of mitral regurgitation recurrence after mitral valve repair compared with posterior leaflet-related lesions. Both edge-to-edge (E-to-E) and neochordal repair, associated with ring annuloplasty, have been used in our institution to address isolated anterior mitral leaflet lesions. The aim of this study was to compare the clinical and echocardiographic long-term results of those two approaches for isolated anterior mitral leaflet lesions by means of a propensity match analysis.

**METHODS:**

An institutional database retrospective review within the time-frame 2000 to 2021 was carried out. The Kaplan–Meier method and cumulative incidence function were employed. Cox regression was used to identify the risk factor for mortality during the follow-up.

**RESULTS:**

The estimated freedom from reoperative mitral valve surgery at 20 years was 78% in the E-to-E group and 64% in the neochordae group (*P* = 0.032). The longitudinal analysis performed to analyse the mitral regurgitation recurrence rate showed a higher rate of mitral regurgitation ≥3+ recurrence in the neochordae group at 5 (5.1% vs 8.7%), −10 (8.2% vs 13.2%), and 15 years (8.8% vs 16.5%) (*P* < 0.001).

**CONCLUSIONS:**

Isolated anterior leaflet pathology can be effectively treated with E-to-E or neochordal repair and ring annuloplasty. In our series, clinical and echocardiographic results were better in E-to-E group. The excellent durability of this technique up to 20 years of follow-up, together with its simplicity and reproducibility, confirms the role the E-to-E techniques as an excellent treatment option for severe mitral regurgitation due anterior mitral leaflets lesions.

## Graphical Abstract

**Figure:**
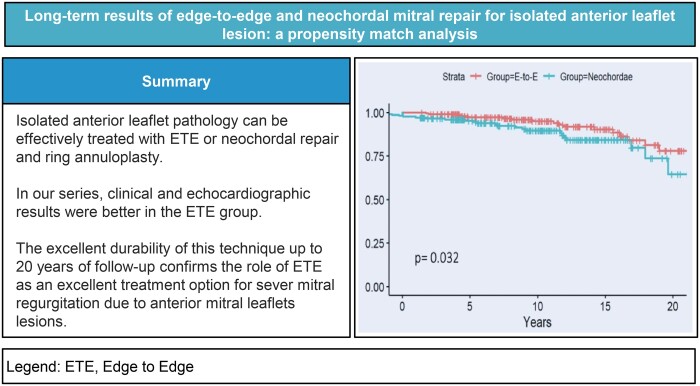

## INTRODUCTION

Nowadays mitral regurgitation (MR) for degenerative disease is routinely treated with valve repair (MVr) with excellent short- and long-term results [1, 2], but inferior outcomes after anterior mitral leaflet (AML)-related lesion repair compared with isolated posterior leaflet defect repair have been reported [3–5]. These worse results may be related to the increased technical challenges in performing anterior leaflet repair [6, 7] and less familiarity or reproducibility with AML repair due to the lower incidence [8] of related pathology.

In the early era of MVr, resection-based techniques, chordal shortening or transposition, and papillary muscle repositioning were frequently used to address AML lesions [9, 10], but in the current era, they are rarely used and have been overtaken mainly by neochordal repair. In our institution, artificial chordae and edge-to-edge (E-to-E), associated with ring annuloplasty, are routinely employed in this context. With this study, we aimed to compare the clinical and echocardiographic long-term results of those two repair techniques for isolated AML lesions. In order to minimize the differences at baseline between the two groups, a propensity match analysis was carried out. Nevertheless the hypothesis would be the difference in outcomes between the two procedures.

## MATERIALS AND METHODS

### Ethical statement

The San Raffaele Hospital Institutional Ethic Committee approved this study on mitral regurgitation and tricuspid insufficiency 125/INT/2022 and waived individual consent for this retrospective analysis.

### Study population and follow-up

A retrospective review of our institutional database was carried out querying all patients who had undergone MVr for isolated AML lesions by means of the E-to-E technique or neochordal implantation associated with ring annuloplasty, within the time frame January 2000–December 2021. We included all patients with lesion of the AML. During the entire time frame the only two techniques used to address lesions of the anterior leaflet have been the E-to-E and the neochordal repair. This last group represents the study cohort. Patient charts were analysed to obtain details about pre-operative characteristics, intraoperative variables, and in-hospital outcomes. To mitigate the differences at baseline between the two groups a propensity match analysis was then carried out. Survival and echocardiographic follow-up were carried out by querying the informatic hospital system for outpatient visits and echocardiographic examinations. If follow-up information was not present in the hospital system, patients and the referring cardiologists were reached via phone call and asked to provide all echocardiographic examinations performed. The cause of death was determined by death certificates or information from the physician who was caring for the patient at that time. The Ethical Committee approved the study and waived the individual informed consent for this retrospective analysis.

### Surgical details and echocardiographic analysis

The great majority of the operations were performed through a conventional midline sternotomy, while the remaining underwent minimally invasive MVr through an anterolateral mini-thoracotomy. Intermittent antegrade cold blood cardioplegia or a single-dose Custodiol crystalloid cardioplegia was used according to the operating surgeon’s discretion. A conventional left atrial incision was used to approach the mitral valve which was systematically assessed to identify location and extension of the AML lesions. The choice of MVr technique used was also left to the operating surgeon’s decision. When an E-to-E MVr was selected a 4.0 or 5.0 polypropylene buttress suture followed by a continuous running suture, suturing the free edge of the prolapsing anterior leaflet to the free edge of the posterior leaflet, was used as previously described [11]. Annuloplasty was then added to complete the MVr in all patients. In case of marked reduction of the valve area, a Hegar dilator was used to exclude mitral stenosis. A global MV area of 2.5 cm^2^ was considered satisfactory for normal size patients (BSA (Body Surface Area), under 1.8 for male and 1.7 for female). When a neochordal repair technique was selected, both hands adjusted 4–0 Gore-tex suture (94; 33%) and pre-formed chordal loops (189; 67%) were employed. In the first part of the study period, hand-adjusted Gore-tex neo-chordae were usually employed, while in more recent years the preformed chordal loops were preferred. Also in this case, annuloplasty was added to complete the MVr in all patients.

Regardless the technique employed, the valve was carefully re-analysed with TEE after weaning from CPB to assess its competence and measure the valve area (particularly for patients who underwent E-to-E repair), usually by a planimetric method using the trans-gastric short-axis view.

The degree of MR was semi-quantitatively measured using Doppler colour-flow imaging and defined as mild (1+/4+) if the percentage of the left atrial area subtended by the MR jet was 1–15%, moderate (2+/4+) if it was 16–35%, moderate to severe (3+/4+) if between 36% and 55% and severe (4+/4+) if >55%. Moreover, the vena contracta width at the narrowest portion of the regurgitant jet was used [12].

### Statistical analysis and propensity score matching

Categorical data were described as absolute and percentage (%) values. Continuous normally distributed variables were expressed as mean  ± standard deviation, while continuous non-normally distributed variables were reported as median and [25th percentile; 75th percentile]. The Shapiro–Wilk test was employed to assess normal distribution. Primary end-points were mortality (overall and cardiac), REDO for severe MR and MR recurrence. The Kaplan–Meier method was employed to estimate survival during the follow-up.

To identify risk factors for mortality during the follow-up, univariate and multivariable Cox regression was employed. The proportional hazards assumption for each covariate was checked by test based on scaled Schoenfeld residuals. Cumulative Incidence Function using death as a competing risk was used to estimate cardiac death and REDO rate for severe MR. The non-parametric Pepe-Mori test was used to make inter-group comparison. The Fine and Gray model for competing risk analysis was employed to evaluate the predictors of cardiac death with death from other reasons as a competing risk, and the REDO rate with death from any reason as a competing risk. Covariates with a *P*-value of <0.1 at univariate analysis were included in the multivariable model.

Finally, to describe the time course of MR recurrence during the follow-up, we performed a longitudinal analysis using generalized estimating equations with a random intercept for correlated data.

**Figure 1:**
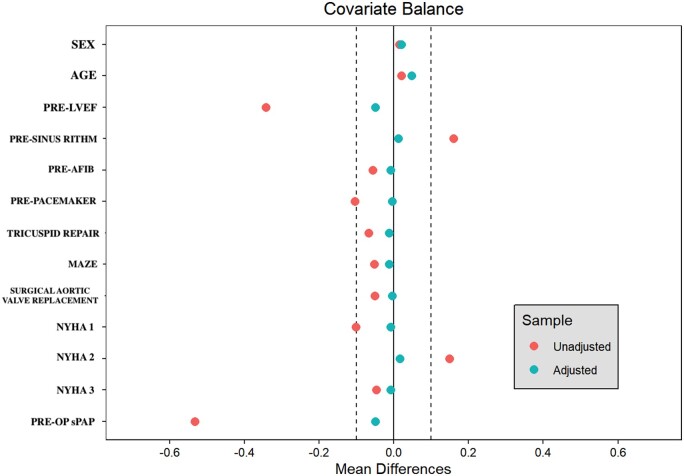
Love plot displaying covariate balance pre- and post-matching. Vertical-dotted lines at ±0.25 indicate the acceptability bounds. After matching all variables stand within the acceptability threshold.

Propensity scores were estimated through logistic regression using the following preoperative variables: age, sex, ejection fraction, preoperative rhythm, New York Heart Association (NYHA) class, preoperative pulmonary pressure, association with tricuspid valve repair, association with atrial fibrillation ablation and association with aortic valve replacement. Patients were matched 1:1 with 0.2 standard deviation calipers using 1- to-1 matching without replacement. Covariate balance was assessed by standardized mean differences, with less than 10% considered acceptable for each covariate after matching was performed. Propensity score distribution and overlapping were displayed with a Love plot showing changes in standardized mean differences before and after matching (Fig. 1) [13].

Risks were reported as hazard ratios along with their 95% confidence intervals (CIs). A P value <0.05 was considered significant.

All analyses were performed using R statistical software (version 4.0.4; https://cran.r-project.org/index.html). The R package MatchIt was used to implement propensity score matching. The R packages survival and cmprsk were used to perform survival and competing risk analyses.

## RESULTS

### Patient characteristics, intraoperative variable and in-hospital results

In the selected timeframe, 5872 patients underwent MVr at our institution: 4121 patients (70.2%) presented a lesion of the posterior leaflet, 1147 (19.5%) had a bi-leaflet prolapse, and an AML lesion was present in the remaining 604 (10.2%). Of these, before matching, 421 underwent MVr with an E-to-E technique and in the remaining 283, a neochordal repair was employed. Patient characteristic of the two groups before matching is listed in Table 1. Patients who underwent mitral valve replacement were excluded. Before patient data matching, associated tricuspid valve repair and atrial fibrillation ablation were significantly more common in the E-to-E group possibly indicating a preference for this technique when a longer cross-clamp time was predicted. The mean ring size used was 35 mm, and the majority were Tailor (Abbott) and semi-rigid Seguin complete ring (Abbott) (70%). After matching, Tailor (Abbott) were 51.7% and semi-rigid Seguin complete ring (Abbott) in 12.6%. Conventional midline sternotomy was performed in 546 (90%), while the remaining 58 patients (10%) underwent minimally invasive MVr through an antero-lateral mini-thoracotomy. A second cross-clamp for suboptimal repair with significant residual MR was necessary in 32 patients (5%), of those, 25 were in the chordal repair group and 7 were in the E-to-E group.

**Table 1:** Unmatched population

	Edge-to-edge	Neochordae	*P-value*
Patients	421	283	
Age (IQR)	58 (45–68)	58 (46–66)	*0.995*
Female (%)	105 (35%)	88 (36%)	*0.797*
Euroscore II	2.33 (1.23–2.38)	2.33 (1.23–2.38)	*0.94*
NYHA class			
I	61 (19 %)	32 (11%)	
II	217 (68 %)	216 (77%)	
III/IV	43 (13 %)	35 (12%)	*<0.001*
LVEF (IQR)	60% (56–65)	55% (55–60)	*<0.001*
Atrial fibrillation	82 (25%)	30 (10%)	<0.001
sPAP (IQR) mmHg	45 (40–50)	35 (35–45)	*<0.001*
Pre-LAD	45 (41–46)	45 (41–48)	*0.087*
Pre-LVEF	59 (56–60)	60 (59–63)	
Pre-RV dysfunction	1 (0.2)	1 (0.4)	*1*
Associated procedure			
Tricuspid valve repair	68 (21%)	32 (11%)	<0.001
Atrial fibrillation ablation	53 (16%)	18 (6%)	<0.001
Aortic valve replacement	22 (7%)	10 (4%)	0.005
Ring diameter, mm (IQR)	35 (32–36)	35 (33–36)	0.661
CEC mean (min)	72 (59–86)	80 (59–85)	0.106

CEC: circulatory ecxtracorporeal circulation; IQR: interquartile range; LAD: left atrial diameter; LVEF: left ventricle ejection fraction; n: number; NYHA: New York Heart Association; PAPs: systolic pulmonary arterial pressure; RV: right ventricle.

In the E-to-E group, two patients (0.5%) died in-hospital; the cause of death was low cardiac output syndrome in two and septic shock in one. Similarly, in the neochordae group, two patients (0.7%) died in-hospital. In the remaining patients, the post-operative course was rather smooth. Low cardiac output syndrome developed in 42 patients, 35 (2%) in the E-to-E group and 7 (2.5%) in the neochordal group. Re-exploration for bleeding was necessary in 13 patients, five in the E-to-E group (2%) and 8 (3%) in the neochordal group. Neurological deficit including stroke and transitory ischaemic attack was present in two patients, 1 patient in the E-to-E group (0.2%) and one in the neo-chordal repair group (0.4%). At hospital discharge, all hospital survivors underwent transthoracic echocardiography. MR was absent or mild in 480 (97%) patients and moderate in 13 (3%) patients in the E-to-E group. Similarly, in the neochordal group, MR was absent or mild in 279 (98%) patients and moderate in the remaining four patients (1.5%). Post-operative mean gradient was 2.5 mmHg (2.08).

The propensity matching yielded 242 E-to-E repairs and 242 neochordal repairs. Matched groups were well-balanced with standardized mean differences of <10% for each covariate (Fig. 1; Table 2). In the matched population, the median age was 56 in both groups [interquartile range (IQR) 44–66 and 45–63 in the E-to-E and in the neochordae group, respectively]. The median left ventricle ejection fraction was 60% (IQR 55–62) in the E-to-E group and 55% (IQR 55–60) in the neochordal group, with a standardized mean difference of 0.04 (Supplementary Material, Fig. S1).

**Table 2:** Matched population

Characteristic	Edge-to-edge	Neochordae	*P*-value	SMD
Patients number	242	242		
Age, median (IQR)	56 (44–66)	56 (45–64)	*0.910*	*0.046*
Gender: female, *n* (%)	87 (36%)	92 (38%)	*0.706*	*0.043*
Euroscore II	2.33 (1.23–2.38)	2.33 (1.23–2.38)	*0.751*	*0.079*
NYHA class				
I	22 (9%)	20 (8%)		
II	192 (79%)	196 (81%)		
III/IV	28 (11%)	26 (11%)	*0.900*	*0.042*
LVEF, median (IQR)	60% (55–62)	55% (55–60)	*0.002*	*0.041*
Atrial fibrillation, *n* (%)	25 (10%)	23 (9%)	0.895	0.043
PAPs (mmHg), median (IQR)	45 (35–45)	42 (35–45)	0.055	*0.048*
Pre-LAD, median (IQR)	45 (41–46)	45 (41–48)	0.465	0.039
Pre-LVEF, median (IQR)	59 (56–60)	60 (59–63)	*<0.001*	0.125
Pre-RV dysfunction	1 (0.4)	1 (0.4)	1	0
Associated procedure, *n* (%)				
Tricuspid valve repair	29 (12)	26 (11)	0.775	0.039
Atrial fibrillation ablation	25 (10)	22 (9)	0.759	0.042
Aortic valve replacement	5 (2)	4 (2)	1.00	0.031
Ring diameter (mm), median (IQR)	35 (31–35)	35 (32.25–35)	0.694	0.024
CEC mean (min), median (IQR)	72 (58–85)	80 (59–85)	0.076	0.129

CEC: Circulatory Ecxtracorporeal Circulation; IQR: interquartile range; LAD: left atrial diameter; LVEF: left ventricle ejection fraction; NYHA: New York Heart Association; PAPs: Systolic Pulmonary Arterial Pressure; RV: right ventricle.

#### Survival analysis

**Figure 2:**
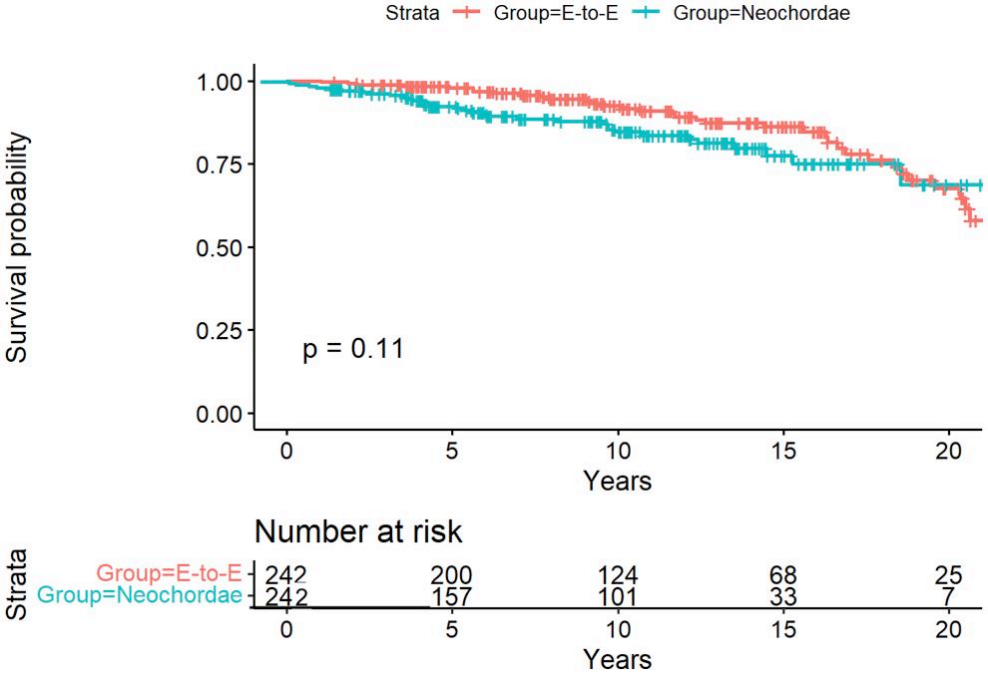
Estimated survival using the Kaplan–Meier method. At 20 years, the estimated survival was 67.6% ± 5.8, 95% CI [57–80] in the E-to-E group and 68.8% ± 7.3, 95% CI [55–84] in the neochoardae group. During the whole follow-up no significant difference in the estimated survival was noted, *P  *= * *0.11.

Clinical and echocardiographic follow-up were 94% complete (28 patients were lost to follow-up); the median follow-up was 10.6 years [IQR 7.3–13.4], maximum: 21 years. Late death occurred in 53 patients in the matched groups. The estimated survival at 5 years, using the Kaplan–Meier method, was 97.3 ± 1.1, 95% CI [95–99] and 95.3% ± 1.5, 95% CI [92–98] in the E-to-E and neochordal groups, respectively (Fig. 2). At 10 and 15 years, the estimated survival was 95.0% ±1.7, 95% CI [91–98] and 90.3% ± 2.8, 95% CI [84–96] in the E-to-E group and it was 89.5 ± 2.5, 95% CI [84–94] and 84.2% ± 3.5, 95% CI [77–91] in the neochordal group. Finally, at 20 years the estimated survival was 67.6% ± 5.8, 95% CI [57–80] in the E-to-E group and 68.8% ± 7.3, 95% CI [55–84] in the neochoardae group.

The Cox regression identified pre-operative pulmonary artery systolic pressure < 25 mmHg as the only significant risk factor (hazard ratio [95% CI]: 0.88 [0.84–0.92], *P  *< * *0.001) for mortality during the follow-up (Table 3). Concerning the clinical condition at the last contact, the majority of the patients were in NYHA class II in both groups: 84% in the E-to-E group and 83% in the neochordal group.

**Table 3:** Predictors of long-term mortality (Cox regression)

Variables	ln (HR)	HR	95% CI		*P*-value
			LB	UB	
Neochordae group	0.49	1.63	0.98	2.71	0.057
Male	−0.01	0.99	0.60	1.64	0.973
Age	0.00	1.00	0.98	1.01	0.870
EF	−0.01	0.99	0.95	1.03	0.580
Atrial fibrillation	−0.12	0.89	0.37	2.12	0.787
Tricuspid valve repair	−0.42	0.65	0.25	1.72	0.390
Atrial fibrillation ablation	0.45	1.56	0.71	3.41	0.264
Aortic valve replacement	−0.14	0.87	0.12	6.55	0.896
NYHA (class II)	0.47	1.60	0.60	4.26	0.346
NYHA (class III)	0.32	1.37	0.41	4.54	0.605
PAPs < 25 mmHg	−0.13	0.88	0.84	0.92	<0.001
MR grade at discharge	−0.05	0.95	0.55	1.65	0.863

HR: hazard ratio; LVEF: left ventricle ejection fraction; MR: mitral regurgitation; NYHA: New York Heart Association; PAPs: systolic pulmonary arterial pressure.

Of the 53 deaths that occurred during the follow-up, only 12 were cardiac deaths. At 10 years, the Cumulative Incidence Function for cardiac death, with non-cardiac death as a competing event, was 3.3  ±  1.93%, 95% CI [1.09–9.95] in the E-to-E group, and it was 12.8 ± 6.93%, 95% CI [2.03–17.9] in the neochordae group; at 15 years, it was 11.2  ±  7.57%, 95% CI [2.08–14.05] in the E-to-E group, and it was 12.8  ±  6.93%, 95% CI [2.03–17.9]. Using the Fine & Gray model, NYHA class was highlighted as a significant risk factor for cardiac death (NYHA class I vs NYHA class III: *P*  <  0.001; NYHA class II vs NYHA class III: *P*  =  0.008) in the long term.

#### REDO rate and mitral regurgitation recurrence

**Figure 3:**
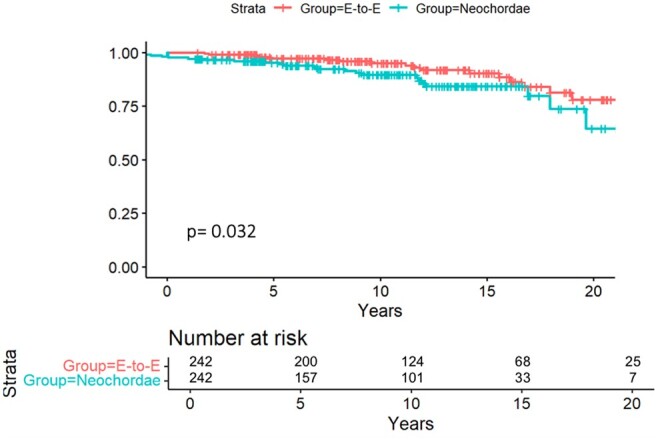
Estimated freedom from REDO for mitral valve surgery using the Kaplan–Meier method. At 20 years, the freedom from REDO was 78% in the E-to-E group and 64% in the neochoardae group with a statistically significant difference (*P* = 0.032).

**Figure 4:**
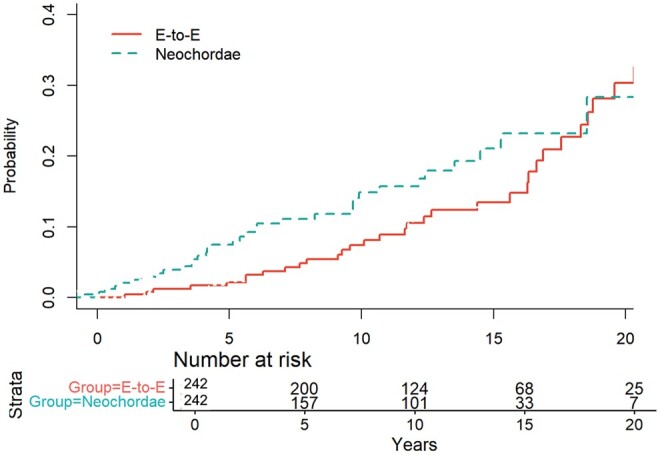
Cumulative incidence function (CIF) for REDO for mitral valve replacement with death for any reason as competing event. At 10 the CIF for REDO for mitral valve replacement with death as competing event was 4.1 ± 1.63%, 95% CI [1.09–7.65] in the E-to-E group and it was 8.6 ± 3.43%, 95% CI [5.12–11.65] in the neochordal group. At 20 years, it was 15.6% ± 5.21%, 95% CI [10.31–20.65] in the E-to-E group and 26.7% ± 6.11%, 95% CI [19.21–32.56] in the neochordae group with a statistically significant difference *P* = 0.038).

During the study period, mitral valve replacement for severe MR was necessary in 40 patients (8.2%). The estimated freedom from reoperation for significant mitral valve dysfunction at 20 years was 78% in the E-to-E group and 64% in the neochordae group with a statistically significant difference (*P*  =  0.032) (Fig. 3). At 10 years, the Cumulative Incidence Function for reoperation for mitral valve replacement with death as a competing event was 4.1  ±  1.63%, 95% CI [1.09–7.65] in the E-to-E group, and it was 8.6 ± 3.43%, 95% CI [5.12–11.65] in the neochordal group. At 20 years of follow-up, it was 15.6% ± 5.21%, 95% CI [10.31–20.65] in the E-to-E group and 26.7% ± 6.11%, 95% CI [19.21–32.56] in the neochordae group (Fig. 4). Using the Fine & Gray model, no significant risk factors for reoperation were identified.

The longitudinal analysis performed by means of the generalized estimating equations showed a significant (*P*  <  0.001) increase in MR ≥ 3+ during the follow-up. At 5 years, the predicted rate of MR ≥ 3+ recurrence was 5.1% in the E-to-E group versus 8.7% in the neochordal group; at 10 years, it was 8.2% in the E-to-E group versus 13.2% in the neochordal group, and finally at 15 years, it was 8.8% in the E-to-E group versus 16.5% in the neochordal group. At the multivariable analysis, no significant risk factor for MR  ≥  3+ recurrence was found. At the last echocardiogram, the mean mitral valve area was 2.9  ±  0.4 cm^2^ and the mean gradient was 3.4  ±  1.1  mmHg.

## DISCUSSION

The main findings of our study are as follows:

1. Isolated AML pathology can be effectively treated with E-to-E or neochordal repair and ring annuloplasty.
2. At very long term (20 years), clinical and echocardiographic results were better in the E-to-E group.

Although similar results have been recently reported in a meta-analysis [14], the importance of the present study lies in the duration of the follow-up, which extends up to 21 years. To the best of our knowledge, a comparison of these two techniques for isolated AML lesions has never been reported with such long-term outcomes and follow-up time-frame.

Degenerative mitral valve disease affecting exclusively the AML is less frequent than posterior or bi-leaflet disease [8], and multiple large series have suggested compromised MVr durability for this entity [3–5]. From an anatomical perspective, the AML is characterized by a lesser degree of redundancy compared to the posterior leaflet despite its larger surface area. The number of AML-related chordae is lower compared to the posterior leaflet, and most of them are concentrated on the leading edge of the leaflet itself. In addition, investigators have observed differences in the forces applied to the leaflets during left ventricular contraction [15] which have been proposed to account for the reported inferior durability of anterior leaflet defect repair compared with that of the posterior leaflet. Moreover, the Cleveland Clinic group reported a linearized annual risk of reoperation of 1.64% for AML repair, significantly greater than the 0.5% annually for posterior leaflet repair [5]. These findings have prompted many surgeons to approach AML degenerative disease with a lower threshold for replacement over repair, and even at valve centers of excellence, repair rates for anterior degenerative disease have been substantially lower than for posterior MV leaflet disease [16].

Neochordae-based repair for AML lesion was originally described in 1989 and, since then, has gained increasing acceptance in the surgical community. However, many surgeons do not feel comfortable with chord sizing, and, due to the increased forces on the AML during ventricular contraction, usually a higher number of neochordae is often used compared to PML repair, inevitably increasing the complexity and duration of the operation [4]. Moreover, the subsequent left ventricular reverse remodelling after MR correction does influence the length of the implanted neochoardae potentially resulting in recurrent MR due to AML re-prolapse [17].

Our group previously reported the long-term results of the E-to-E technique for isolated AML lesions with a very low rate of MR recurrence and re-operation [18], making the results of the current study not particularly surprising for us. Nevertheless, in the cardiac surgery community, some reluctance to employ the E-to-E repair still exists, despite the increasing evidence also provided by similar percutaneous MVr techniques [19, 20]. This study and other available data do not support this concern, both in posterior, bi-leaflet and anterior leaflet lesions [14, 21–23].

In conclusion our study suggests that E-to-E repair for the treatment of isolated AML lesions may be associated with greater freedom from MR recurrence than neochordae repair. Moreover, compared to neochordal repair, the E-to-E technique is undoubtedly more reproducible and easier to perform compared to other MVr techniques.

### Limitations

The main limitations of our study are related to its retrospective nature. Moreover, although mitral stenosis at rest was never detected, exercise echocardiography to exclude this event under stress was not performed in this specific sub-group of patients. Not all the echocardiograms have been performed at our institution, but the echocardiographic data are fairly complete to provide solid and comprehensive conclusions about the rate of MR recurrence. Finally, we were not able to collect detailed information about the arrhythmia treatment and hypertensive therapy because these decisions were left to the caring physician.

## CONCLUSION

Isolated AML pathology can be effectively treated with E-to-E or neochordal repair and ring annuloplasty. In our series, clinical and echocardiographic long-term results were better with the E-to-E technique which has been the preferred approach at our institution in the last 20 years. The excellent durability of this technique up to 20 years of follow-up, together with its simplicity and reproducibility, confirms the role of the E-to-E techniques as an excellent treatment option for severe MR due to AML lesions.

## Supplementary Material

ezae435_Supplementary_Data

## Data Availability

The data underlying this article will be shared on reasonable request to the corresponding author.

## Notes

Presented EACTS Annual Meeting 2023, Vienna

## ABBREVIATIONS

AML: Anterior mitral leaflet
CI: Confidence interval
E-to-E: Edge-to-edge
IQR: Interquartile range
MR: Mitral regurgitation
MVr: Mitral valve repair
NYHA: New York Heart Association
