# Supplementary material for: Long-term results of edge-to-edge and neochordal mitral repair for isolated anterior leaflet lesion: a propensity match analysis
Source: Eur J Cardiothorac Surg. 2024 Dec 3;66(6):ezae435. doi: 10.1093/ejcts/ezae435 (PMC11646568; doi:10.1093/ejcts/ezae435)
Supplement: ezae435_Supplementary_Data [file ezae435_supplementary_data.docx]

**Supplementary figure 1:**

The Curve shows the pre- and post-matching distribution of the two techniques studied across the study period.
